# Semantic Integration of Cervical Cancer Data Repositories to Facilitate Multicenter Association Studies: The ASSIST Approach

**DOI:** 10.4137/cin.s963

**Published:** 2009-02-03

**Authors:** Theodoros Agorastos, Vassilis Koutkias, Manolis Falelakis, Irini Lekka, Themistoklis Mikos, Anastasios Delopoulos, Pericles A. Mitkas, Antonios Tantsis, Steven Weyers, Pascal Coorevits, Andreas M. Kaufmann, Roberto Kurzeja, Nicos Maglaveras

**Affiliations:** 1 Department of Obstetrics and Gynecology, Papageorgiou General Hospital, Aristotle University of Thessaloniki, Greece; 2 Lab of Medical Informatics, Medical School, Aristotle University of Thessaloniki, Greece; 3 Department of Electrical and Computer Engineering, Faculty of Engineering, Aristotle University of Thessaloniki, Greece; 4 Informatics and Telematics Institute, Centre for Research and Technology Hellas, Greece; 5 Department of Obstetrics and Gynecology, Faculty of Medicine and Health Sciences, Ghent University, Belgium; 6 RAMIT vzw, c/o Department of Medical Informatics and Statistics, Ghent University Hospital, Ghent, Belgium; 7 Gynäkologie, Charité-Universitätsmedizin Berlin, Germany

**Keywords:** association studies, cervical cancer, coding systems, data unification, semantic integration

## Abstract

The current work addresses the unification of Electronic Health Records related to cervical cancer into a single medical knowledge source, in the context of the EU-funded ASSIST research project. The project aims to facilitate the research for cervical precancer and cancer through a system that virtually unifies multiple patient record repositories, physically located in different medical centers/hospitals, thus, increasing flexibility by allowing the formation of study groups “on demand” and by recycling patient records in new studies. To this end, ASSIST uses semantic technologies to translate all medical entities (such as patient examination results, history, habits, genetic profile) and represent them in a common form, encoded in the ASSIST Cervical Cancer Ontology. The current paper presents the knowledge elicitation approach followed, towards the definition and representation of the disease’s medical concepts and rules that constitute the basis for the ASSIST Cervical Cancer Ontology. The proposed approach constitutes a paradigm for semantic integration of heterogeneous clinical data that may be applicable to other biomedical application domains.

## 1. Introduction

Lack of uniformly structured data affects many areas of biomedical research, including drug discovery, systems biology, and individualized medicine, all of which rely heavily on integrating and interpreting data sets produced by different experimental methods at different levels of granularity, for which both the discovery and characterization is extremely difficult.^1^ The heterogeneity and “isolation” of biomedical resources has been identified as the key research challenge,^2^ towards the realization of translational medicine.^3^

Genetic association studies constitute a significant scientific approach that may lead to a more comprehensive and holistic insight on the origin of complex diseases,^4^ aiming to identify association between one or more genetic variants (e.g. polymorphisms) and a trait, which might be some quantitative characteristic, a discrete attribute, or a disease.^5^ A genetic variant is genotyped in a population for which phenotypic information is available (such as disease occurrence, or a range of different trait values). If a correlation is observed between genotype and phenotype, there is an association between the variant and the disease or trait.^6^

Although the number of studies elaborating on phenotype-genotype associations for common diseases is rapidly increasing, several studies show variation in the underlying association between genotype and outcome among the populations studied, resulting in questionable findings. It is evident that reliable association studies require large sets of patient phenotypic and genotypic data, all provided in a structured format.^7^ Thus, methodologies for data and system interoperability, assisted by the appropriate technologies, will alleviate the problems mentioned above. While standardisation of phenotypic data and clinical practices seems unrealistic, semantic annotation and transformations to widely agreed classification schemes may provide workable solutions.

In this regard, Semantic Web technologies,^8^ which are based on common formats that support aggregation and integration of data drawn from diverse sources, seem a key-enabler towards navigation and meaningful use of digital resources by employing automatic processes. The benefits promised by the Semantic Web include aggregation of heterogeneous data using explicit semantics, simplified annotation and sharing of findings, the expression of rich and well-defined models for data aggregation and search, easier reuse of data in unanticipated ways, and the application of logic to infer additional insights.^9^ The potential of this technology endeavor has been particularly highlighted in biomedicine with initiatives such as the Semantic Web Health Care and Life Sciences Interest Group (HCLSIG) established by W3C (World Wide Web Consortium), which is chartered to explore and support the use of Semantic Web technologies to improve collaboration, research and development, and innovation in the information ecosystem of the health care and life science domains. Especially in biomedicine, it is envisioned that the use of Semantic Web technologies will improve the productivity of research, help raise the quality of health care, and enable scientists to formulate new hypotheses inspiring research based on clinical experiences.

Ontologies, considered as the backbone of the Semantic Web, play an important role in biomedical research through a variety of applications.^10^ They provide the controlled vocabulary required for the annotation of biological datasets, the biomedical literature and patient records, facilitating the retrieval of and, more generally, access to information. Such standardization also facilitates the exchange of information and contributes to semantic interoperability among systems. By providing a representation of a domain, ontologies are also used in the mediation approach to integrating datasets.^11^ Finally, many applications use ontologies as a source of computable domain knowledge, including natural language processing applications and decision support systems.^12^ Ontologies are also critical to hypothesis generation and knowledge discovery in a data-driven approach to biomedical research.^13^

The concept of supporting in a systematic way the conduction of multi-center association studies has been elaborated in ASSIST (Association Studies aSsisted by Inference and Semantic Technologies) by adopting inference and semantic technologies.^14^ ASSIST is an EU-funded research project that aims to facilitate the design and execution of genetic association studies on cervical precancer and cancer (CxPCa). Toward this goal, ASSIST provides the means for seamless integration and virtual unification of distributed and heterogeneous CxPCa data repositories, as well as the underlying mechanisms to undertake the entire process of expressing and statistically evaluating medical hypotheses based on the collected data, in order to generate medically important associations. ASSIST aims to be used primarily by biomedical researchers for the identification of new markers of risk, diagnosis and prognosis, and possibly treatment of cervical precancer and early cancer via the implementation of case-control association studies.

Cervical cancer is an important cause of women mortality throughout the world. Current evidence suggests that specific subtypes of a certain virus, namely, the Human Papilloma Virus (HPV), cause genital infection that predisposes to cervical cancer development later in life.^15^ Under research are other cofactors of cervical precancerous lesions. Tobacco use, number of sexual partners, number of deliveries, use of hormonal contraception, are characteristics that can put individuals at risk for CxPCa.^16^ Moreover, genetic polymorphisms, such as MethyleneTetraHydroFolate Reductase (MTHFR), p53 codon 72, Glutathione-S-Transferase (GST) T1, GST M1, and cytochrome P1 (CYP1) A1, are currently under investigation about their role in the pathogenesis of this disease.^17^–^21^

The current paper presents the knowledge elicitation approach followed towards the definition and representation of medical concepts related to diagnosis and disease severity that constitute the basis for the ASSIST CxPCa semantic model, emphasizing on the data unification procedure applied in three busy clinical sites across Europe. The paper is structured as follows. First, the origin and nature of the CxPCa data repositories considered in ASSIST are described. Then, the major CxPCa semantic medical concepts elaborated in the knowledge engineering phase of the project are presented. The common coding towards the semantic unification of CxPCa data repositories is then introduced, as far as the major clinical interventions related to CxPCa are concerned. The approach followed to infer the disease severity based on semantic rules associating the clinical findings of the abovementioned medical interventions is then elaborated, while aspects related to the implementation of the semantic model and an example usage scenario are presented. Finally, a discussion on related works as well as on the virtue of the proposed approach and its potential conclude the paper.

## 2. Materials and Methods

### 2.1. Cervical cancer data repositories

The ASSIST project aims to help medical researchers and clinicians to perform association studies with the use of information technologies (IT) within an open, integrated platform, allowing access to existing patient records from three different European clinics. More specifically, ASSIST currently integrates patient data originated from three gynecology clinics located in Belgium (Ghent University Hospital), Germany (Charité Hospital, Berlin) and Greece (Papageorgiou General Hospital, Thessaloniki). Each data repository has its own autonomous schema and captures patient information related to CxPCa, according to the local clinical procedures followed. An ethical committee approach was obtained as appropriately from each site of data collection before the utilization of patients’ data. In addition, those data were anonymised prior to their availability via the ASSIST platform.

Personal data included in the databases were: date of birth, age at presentation, place of living, gravidity, parity, tobacco use (cigarettes per day and smoking years), use of oral contraceptives (years), use of intrauterine contraceptive device (years). Data regarding cervical HPV exposure included the age at the date of 1st HPV testing, whether HPV presence was detected in each visit, whether a low risk or a high risk HPV type was detected, as well as the kind of testing (e.g. PCR, hybrid capture, specific commercial kits, etc.). PAP smears were used for HPV genotyping. Cytology, colposcopy, and—when available—histology results were recorded (details given in the next paragraph). In cases where an intervention (i.e. punch biopsy of the cervix, large loop excision of transformation zone (LLETZ), conectomy or hysterectomy) was necessary, the kind, the date, and the result of the intervention were also available. All data are in form of discrete categorical values, i.e. no vital signal and image data are included.

Furthermore, genetic test results for polymorphisms possibly associated with CxPCa, i.e. p53 codon 72 polymorphisms (arginine/arginine omozygotes, arginine/prolin heterozygotes, prolin/prolin omozygotes), MTHFR polymorhisms (cytocine/cytocine omozygotes, cytocine/thymine heterozygotes, and thymine/thymine omozygotes), GSTT1, GSTM1, and CYP1A1 polymorphisms (wild type, heterozygotes, and null genotype), were also considered when available. For SNP analysis, either PAP smear or blood sample was used.

Data recruitment from the three different busy clinical sites across Europe, increased number of patients enrolled in the project, and virtual unification of these data, are all factors that increase the power and quality of association studies minimizing the limitations of the outcomes. At this point, records from 2,038 patients (more than 3,000 visits) have been made accessible to ASSIST by the three clinics. Using information from these medical records, research studies can be implemented to extract associations between CxPCa and viral, lifestyle and genetic factors aiming to advance knowledge on the origin and progress of the disease.

ASSIST virtually unifies these heterogeneous data sources syntactically, by defining a common data schema, and semantically via a domain ontology for precancerous and cancerous cervical lesions that encapsulates the appropriate medical knowledge to model the disease towards inferring its severity via the available diagnostic information (cytology, colposcopy and histology) and HPV infection results, as well as to express other disease-related information such as putative genetic polymorphisms associated with CxPCa, lifestyle and personal information. The main challenge has been to unify the three types of diagnostic results, coded according to locally used schemes, and infer disease severity expressed in a uniform and medically sound way. Thus, uniform coding has been elaborated for each type of diagnostic test result, so that the underlying heterogeneity in each clinic site is addressed. Interpretation to the limited existing standardised coding schemes is also supported when possible. This uniform classification of exam results is then employed in the context of medical rules for the inference part of the system, so as to identify the disease severity.

### 2.2. Cervical cancer semantic concepts

The typical approach for diagnosis and treatment of precancerous cervical lesions and/or invasive cancer includes anamnesis, routine clinical examination, smear test sampling (cytology), disease-specific clinical examination (colposcopy) and, if clinically necessary, biopsy sampling from suspicious for cancer sites (histology). Contemporary medical knowledge and clinical cost-effectiveness dictate that where cytology and colposcopy are normal, no further interventions are required. On the most of other occasions, especially in the presence of moderate or severe cytological and/or colposcopical abnormalities, histology is mandatory in order to exclude or to verify the disease.^22^ Furthermore, HPV testing may be advised in certain cases and lifestyle related questions may be asked to the patient.

In current clinical practice there is no uniform way of describing cytology, colposcopy and histology findings. It was therefore realized that a need was eminent for common interpretation of similar conditions and diseases, and for common clinical protocols, if possible. The challenge of aggregating medical information lying in disperse databases, though not easy, could enable the reuse and exploitation of existing medical data for clinical and research purposes.

It has to be noted that no genetic factor has been established so far as to be a risk factor for the development of cervical neoplasia; hence, genetic testing is not part of clinical practice for the disease. However, genotype information on certain polymorphisms may exist in medical records as part of research.

### 2.3. ASSIST coding

Towards a common interpretation of the diagnostic test results, a scheme for uniform coding has been defined for the crucial fields of cytology, colposcopy and histology. These test results express the severity of cervical disease although they vary in the level of detail and type of information. ASSIST introduces a uniform coding scheme for all three tests consisting of 4 distinct classes (Table 1): the first one includes test results with normal findings, the second class indicates Low Grade Cervical Intraepithelial Neoplasia (LCIN), the third class indicates High Grade Cervical Intraepithelial Neoplasia (HCIN) and, finally, the fourth class corresponds to Invasive Cervical Cancer. The proposed coding scheme (from now on referred as “**ASSIST coding**”) appears a reasonable option because: (a) it follows a simple index of severity of cervical precancerous disease, (b) it is pragmatic, and (c) it is closely related to clinical decision making and follow-up. More specifically, the ASSIST coding is closely related with the main clinical decisions that a physician has to make during the management of women with normal cervical screening results, precancerous lesions or invasive cancer of the uterine cervix.

In particular, regarding cytology results, there are two classification systems widely used, namely, the Bethesda and the Munich system (an extension of the Papanicolaou (PAP) classification), parallel to the detailed description of findings.^23^–^27^**ASSIST cytology Code 0** includes normal cells, PAP I, PAP II, metaplasia, infection, regeneration, and atrophy. **ASSIST cytology Code 1** includes atypia, koilocytic atypia, HPV alterations, ASC-US, PAP IIw, PAP IIk, PAP III, PAP IIID, mild dyskaryosis, mild dysplasia, LGSIL (Low Grade Squamous Intraepithelial Lesion), CIN1, AGUS, and ACG-NOS. **ASSIST cytology Code 2** includes ASC-H, PAP IIIG, PAP IVa, PAP IVb, moderate dyskaryosis, severe dyskaryosis, moderate dysplasia, severe dysplasia, HGSIL (High Grade Squamous Intraepithelial Lesion), CIN2, CIN3, AIS (Adenocarcinoma in situ), and AGC—favor neoplasia. Finally, **ASSIST cytology Code 3** includes PAP V, cellular changes suggestive of invasive squamous carcinoma, and cellular changes indicative of adenocarcinoma of the uterine cervix and other invasive cancer.

Regarding colposcopy results, **ASSIST colposcopy Code 0** includes within normal limits outcome (normal—metaplasia—infection—atrophy); **ASSIST colposcopy Code 1** includes low grade abnormal findings; **ASSIST colposcopy Code 2** includes high grade abnormal findings; and **ASSIST colposcopy Code 3** includes findings suggesting invasive cancer.^22^

Similarly, for histology results, **ASSIST histology Code 0** includes normal cervical epithelium, metaplasia, infection, hyperplasia, and atrophy. **ASSIST histology Code 1** includes condyloma, coilocytic atypia, atypia, mild dysplasia, low grade dysplasia, and CIN1. **ASSIST histology Code 2** includes moderate dysplasia, CIN2, severe dysplasia, high grade dysplasia, CIN3, CIS (Carcinoma in situ) and adenocarcinoma in situ (AIS). Finally, **ASSIST histology Code 3** includes invasive squamous cell carcinoma (SCC) and adenocarcinoma of the cervix.

Since ASSIST coding consists of 4 classes, it poses limitations when mapping test results expressed with higher level of detail. Unfortunately, there is no standardization related to colposcopy and histology results, therefore, clinics tend to use locally defined coding schemes. However, cytological results may be described using the two established classification systems, namely, Bethesda and Munich, in which case they are preserved by ASSIST. Specifically, if cytological results coming from a specific clinic use either Bethesda or Munich classes (or a subset of them), then ASSIST will keep this classification, in addition to the ASSIST mapping. However, if cytological results are expressed in merged Bethesda or Munich classes, then the ASSIST classification will be used instead. As a result, it is optional to request and retrieve cytological results expressed uniformly either in Bethesda or in Munich (provided that the original records contain the necessary information) or in the ASSIST coding scheme. Tables 2 and 3 depict two examples of cytology results integration. In case 1, the University Hospital of Ghent uses a cytology classification with a subset of distinct Bethesda classes; cytology records are therefore integrated in ASSIST in two ways: i) preserving Bethesda classification and ii) mapped to ASSIST coding. In case 2, however, the Charité Hospital classifies cytology results in merged classes from both the Bethesda and Munich system. Specifically, although most classes are distinct Munich classes, the HSIL class used (which is a Bethesda term) cannot be mapped to a distinct Munich class. In this case, integration is performed using the ASSIST coding. Cytology results can always be mapped to the four distinct ASSIST codes that are related to clinical decision making during management of women with normal cervical screening results, precancerous lesions or invasive cervical cancer.

### 2.4. ASSIST severity index

The amount of the information collected and expressed by using the ASSIST coding, is finally represented by an index, called “**Severity Index**” (SI), which is the equivalent for the existing risk for cervical malignancy of the individual woman. This index is closely related with the main clinical decisions that a physician has to make during the management of women with normal screening results or with already detected precancerous lesions or the cervix. Individuals with normal or within normal limits results, independently of the way they have been obtained (i.e. via cytology, and/or colposcopy, and/or (seldom) histology), are basically discharged from the clinic with instructions for regular follow-up according to the local protocols. These women are essentially healthy individuals and correspond to “**SI = 0**.” Women with results that need to be followed-up earlier than usual and do not mandate ablation or excisional treatment of the altered cervical epithelium (i.e. HPV-related alterations, presence of atypical squamous cells of unknown significance, CIN1, satisfactory colposcopy with low grade abnormal findings and low grade histology in biopsy) are encoded with “**SI = 1**.” Women who cytologically are found to have CIN2–3, women who have satisfactory colposcopy with high grade lesions, and women who had LLETZ or cone biopsy and histologically proven high grade cervical lesions but not stromal invasion are coded with “**SI = 2**.” Finally, women with invasive cervical cancer (squamous cell carcinoma or adenocarcinoma) are coded with “**SI = 3**.”

In order to define the SI based on the results of cytology, colposcopy and histology, all these results have to be first unified and interpreted in the ASSIST coding. Every medical record is essentially associated to a patient **Visit** and may contain one or more examinations. An examination may be any of the diagnostic interventions, namely, cytology, colposcopy, histology and HPV test. The set of examinations that are defined and performed when a medical guideline is followed for a certain patient during a specific time period, constitute a **Case**. Patients, Cases and Visits should be identified in each database. It is clear that the concepts of Patients and Visits already exist in each database; however, the Case identification should also be provided. In this context, the SI is defined per case and is derived from the results of cytology, colposcopy and histology that may be associated with (through the corresponding visits) as follows. ASSIST identifies all cytology examinations in a case and keeps the most severe one. The same applies for colposcopy and histology examinations. Then, the SI of the case is inferred from the final set of the three examinations according to the rules described in Table 4.

Severity Index distinction in 4 classes similar to ASSIST coding, appears to be clinically acceptable because it is closely related to major clinical outcomes and subsequent major clinical decisions. The SI is exclusively depended on the clinical results. Today, the status of a cervix is defined as: a) normal, b) with low grade lesion, c) with high grade lesion or d) with cancer. In this regard, the SI (from 0 to 3) directly corresponds to this stratification, and therefore is considered to be a tool enhanced by clinical significance.

**Figure 1:**
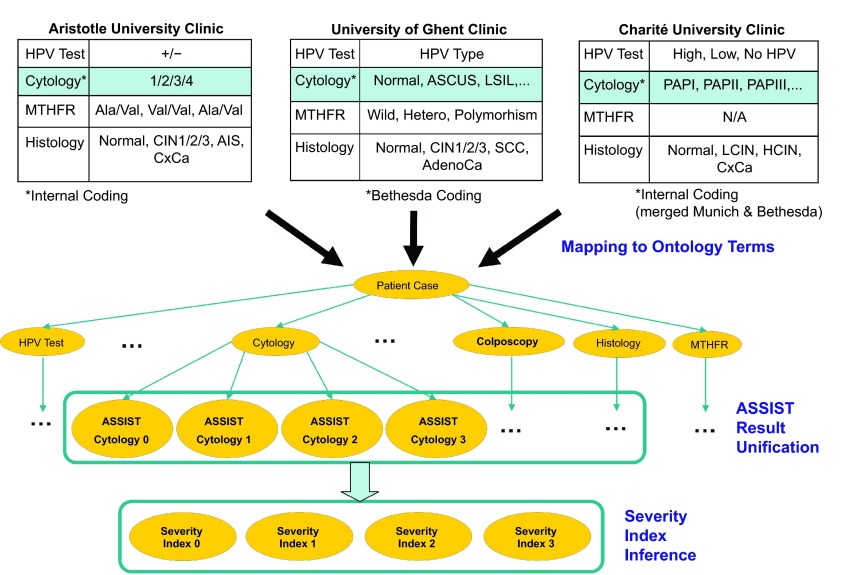
Heterogeneity in biomedical data related to cervical diagnostic examinations in individual patients and their virtual unification.

Figure 1 depicts the overall data unification scheme employed in ASSIST that leads to the identification of the SI for each patient. The abovementioned semantic concepts and rules on CxPCa have been encoded formally and incorporated in the ASSIST Cervical Cancer Ontology that is described in the following.

## 3. Results

### 3.1. ASSIST semantic model

The ASSIST Cervical Cancer Ontology, being the first one to ever semantically model CxPCa concepts, was designed and constructed following the medical knowledge elicitation phase described above. It encapsulates basic EHR (Electronic Health Record) information, examination results and genetic information together with the medical rules defined, so as to enable the mapping of diverse terms originated from each CxPCa data repository into a common schema and finally to infer the SI for CxPCa of each patient.

An interesting property of the ASSIST Cervical Cancer Ontology is that it provides great level of extensibility. The concept hierarchy is expanded from rather generic medical entities to specific CxPCa ones. This way, it can virtually incorporate any cancer domain knowledge and can even be further extended to other medical domains as well. This could provide potential means for representation of diseases and medical concepts and research on interrelation of diseases.

As noted in section 2.4, computation of SI is based in the notion of Case. It is, therefore, natural for the conceptual model that is built around the *Case* concept. This is directly connected with the notions of *Visit, Clinic, Person, Severity Index,* and *Lifestyle Choice* (including *Smoking, Parity,* and *Use of Contraceptives*) and indirectly associated with others like *Diagnostic Examination* (including *Cytology, Colposcopy, Histology* and *HPV Test), Therapeutic Intervention, HPV Vaccination* and *Genetic Polymorphisms*.

Entities constituting the ASSIST Cervical Cancer Ontology are essentially of two types:

–*Generic EHR entities*: These are generic in the sense that they are not CxPCa specific and can be extracted directly from the Hospital Information System. Included here are concepts like Patient, Case, Visit, Clinic, Medical Intervention, Lifestyle Choice etc.
–*CxPCa-specific entities*: These are usually subclasses of the previous ones and are intended to model knowledge useful to describe CxPCa disease and related factors. This type includes Cytology, Colposcopy, Histology, CxPCa Severity Index, as well as HPV and genetic polymorphism information.

**Figure 2:**
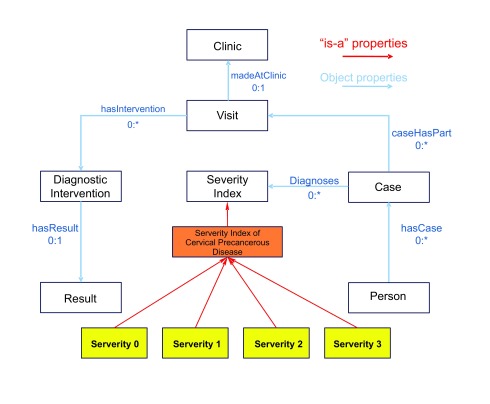
Schematic representation of the basic concepts and their relations in the ASSIST Cervical Cancer Ontology.

Figure 2 depicts the major concepts of the ASSIST Cervical Cancer Ontology, as well as their associations via appropriate properties.

### 3.2. Rules for semantic inference

**Figure 3:**
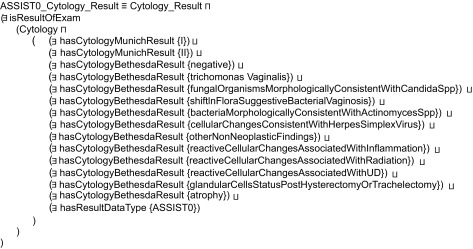
Example restriction rule employed to achieve the semantic unification of cytology results that correspond to findings within normal limits in terms of the ASSIST coding scheme.

**Figure 4:**
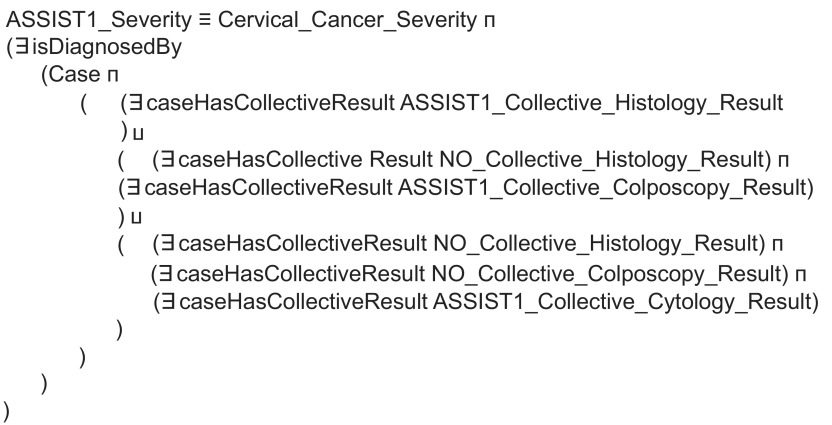
Example restriction rule employed to aggregate unified examination results so as to infer SI = 1 for the corresponding case.

As far as reasoning is concerned, this is based on two types of rules, expressed as Description Logics restrictions,^28^ which produce inferred types for proper characterization of asserted knowledge. The first type of restrictions is basically employed to achieve the semantic unification of examination results in terms of the ASSIST Coding scheme. Figure 3 depicts the example of a restriction employed to infer that a cytology has indicated findings within normal limits, i.e. ASSIST cytology Code = 0, using the rules as defined in Tables 2 and 3. The second type of restrictions aims at the aggregation of unified examination results, to produce verdict on the SI of the corresponding case, as defined in Table 4. Patients categorized as having SI = 1 are inferred by employing the restriction depicted in Figure 4.

### 3.3. Implementation details

The ASSIST Cervical Cancer Ontology was developed in Protégé knowledge modeling tool,^29^ while the knowledge representation language employed was OWL-DL.^30^ Sesame (http://www.openrdf.org/) and OWLIM^31^ were chosen for storage and reasoning, respectively.

Furthermore, a simple medical-researcher oriented ontology editor was developed, in order to ensure easy and secure maintainability and incorporation of new knowledge. This tool permits easy rule insertion and concepts/properties manipulation requiring no knowledge engineering expertise, while assuring that vital concepts and interrelations remain unaffected.

The ASSIST Cervical Cancer Ontology TBox currently consists of 163 classes, 21 object properties, 33 datatype properties and 26 equivalent class axioms, while current instantiation of the ontology ABox contains about 60,000 individuals.

### 3.4. Example usage scenario

**Figure 5:**
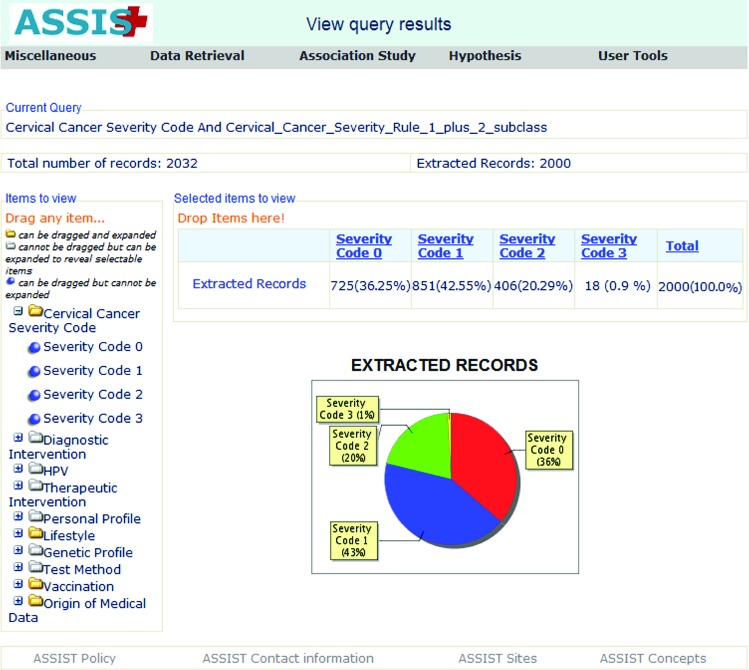
**Viewing all available patients by Severity Index:** On the left, all medical entities related to cervical cancer and pre cancer are displayed in a tree structure. The user can expand the tree items and select the type of information he/she wishes to use as search criteria. In this example, the user has selected Cervical Cancer Severity Code, meaning that all patients, for whom SI can be inferred, will be extracted. The preview of the query is shown on the top of the screen. On the right, the number of extracted records can be viewed both in table and graph format. For 32 patients, no diagnostic information was available; therefore, SI could not be inferred.

Suppose a researcher wishes to investigate whether there is an association between the MTHFR polymorphism and cervical precancerous lesions. While planning the study, he/she consults ASSIST to see, if there are shared data from patients with LCIN, HCIN and healthy patients for whom MTHFR genotype data are also available. The ASSIST User Interface offers the option to use the inferred ASSIST Cervical Cancer SI to express LCIN or HCIN, which is defined on the basis of the medical rules explained in section 2.4. Selecting a query to view all patients by inferred SI, the user can rapidly check the type of patients for whom data are shared through the ASSIST infrastructure (Fig. 5).

**Figure 6:**
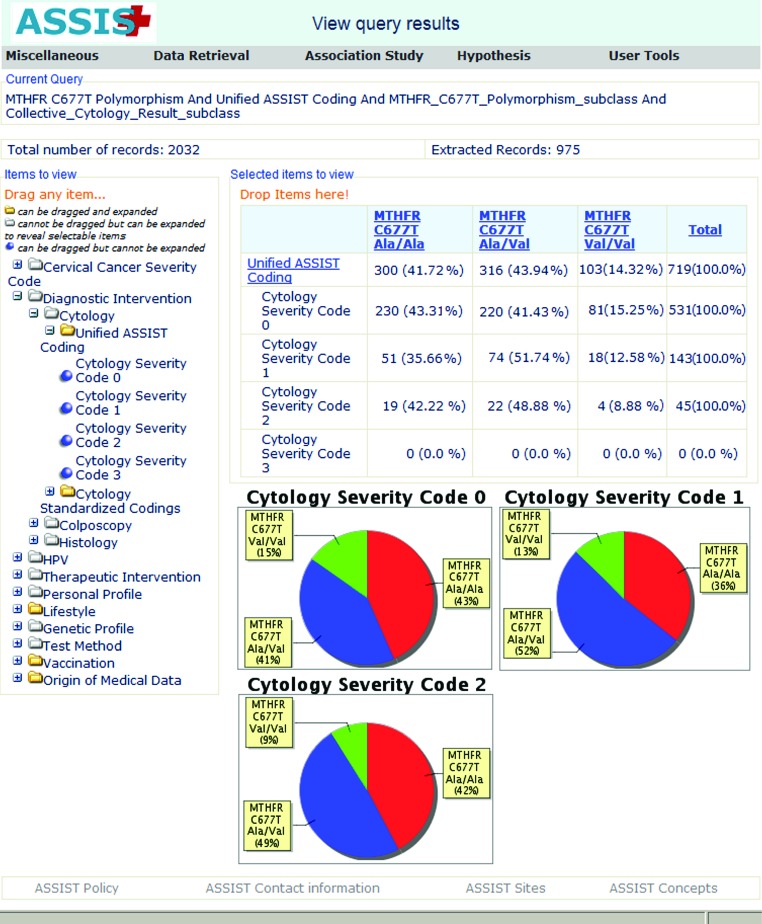
**Viewing the distribution of MTHFR genotypes in patients with different cytology results:** In the current example, the user has selected all patients with data for the MTHFR polymorphism. Then, he/she has selected to view the MTHFR distribution of the three MTHFR genotypes in patients with different cytology results. A preview of the current query is shown on the top of the page. The cytology results are expressed with the unified ASSIST cytology coding. As shown on the top of the screen, from the total of 2,032 patients the requested type of information exists for 975 patients.

The user can express the severity of the disease through the results of cytology or colposcopy or histology or any combination of those. The results of those exams are expressed through the unified ASSIST coding. Using combined queries, the researcher asks for patients with genotypic data for the MTHFR polymorphism and for whom the staging of precancerous lesions is expressed through cytological results coded in the ASSIST unified cytology coding (Fig. 6). The graphical representation of the results indicates that the distribution of the MTHFR genotypes is different in the three stages of the disease. The user can continue with more queries involving additional patient parameters and further statistical tests on the extracted dataset. ASSIST offers a user friendly environment to express queries in a “language” easily understandable by the medical researcher and to also retrieve patient records that may use different internal coding schemes.

## 4. Discussion

Lately, there is an increasing research interest in genetic association studies, emphasizing in quality aspects. The potential of reusing existing patient data can lead to high number of samples, thus, increasing power of the tests, while at the same time reducing the cost for the implementation of such studies.^32^ Several projects are under development, aiming to provide integration tools that will allow accessibility and interpretation to a wealth of clinical records in disperse repositories. For example, i2b2 (https://www.i2b2.org/) is an NIH-funded research project developing a scalable IT framework that will bridge clinical research data and the vast data banks arising from basic science research, in order to better understand the genetic bases of complex diseases. The UK-founded CLEF project (http://www.clinicalescience.org/) focuses on generic methods for the capture, processing, and dissemination of information about cancer patients and their care using EHR data integrated with support for clinical and basic research in the biosciences. It aims to incorporate this information in formal knowledge management systems within a Grid framework. In the same fashion, the EU-funded NeuroWeb project (http://nuke.neurowebkc.eu/) aims to improve healthcare delivery related to neurosciences, achieving knowledge-based, personalized diagnosis and therapy through vertical integration of existing clinical and genetic databases. It stimulates the sharing of knowledge on cerebrovascular diseases using a Web-based platform. The Healthe-Child project (http://www.health-e-child.org/) aims at developing an integrated healthcare platform for paediatrics, providing seamless integration of traditional and emerging sources of biomedical information, primarily focusing on individualized disease prevention, screening, early diagnosis, therapy and follow-up of paediatric heart diseases, inflammatory diseases, and brain tumours. In most of these efforts, semantic technologies have been employed as an enabling technical endeavor for biomedical data integration and knowledge modeling. It has to be noted that, in such efforts, ethical and regulatory issues need also to be addressed in combination with privacy and security aspects.

ASSIST moves in a parallel course with the abovementioned research initiatives, aspiring that its platform will function as an IT tool enabling association studies linked with cervical cancer research by establishing a collaborative environment and allowing any medical group active in this area to use its facilities and/or contribute their own data/results. Cervical cancer, the second most common cancer in women, is recently subject of a tremendous interest by the scientists all over the world, especially because of the new knowledge concerning the causal role of HPV in the pathogenesis of the disease, and the new perspectives of disease prevention through prophylactic vaccination against viral infection. However, the interactions between HPV infection, environmental factors and host genetic background have not been yet fully elucidated and still reflect a field of continuing research interest. To this end, ASSIST aims to offer a new technological solution that will virtually unify multiple patient record repositories (physically located at different laboratories, clinics and/or hospitals) thus, enabling researchers to utilize existing genotypic and phenotypic patient data from several clinics and perform association studies in a low-cost and time-efficient way. Currently, a unified CxPCa data repository has been constructed, as a result of the ASSIST semantic integration methodology described above. With regards to genetic data, the current version of the ASSIST Cervical Cancer Ontology includes genotypic information of polymorphisms suspected to cause cervical cancer. Such data are currently available in research databases and can directly be exploited through the ASSIST platform and produce association study results. As genomic sequencing results will become available in the following years, it is evident that the ASSIST Cervical Cancer Ontology has to be expanded to incorporate such kind of information.

Following a generic design, the ASSIST system may be expanded in terms of its underlying knowledge model, in order to facilitate genetic association studies for other diseases, e.g. colon cancer and cardiovascular diseases. As presented in this paper, in ASSIST the main challenge has been to unify diagnostic results and severity index which are expression of disease severity. The presented approach constitutes a paradigm for semantic integration of heterogeneous clinical data that may be applicable to more diseases. The ultimate goal for ASSIST is to foster the biomedical research community by providing an open, integrated and collaborative framework to facilitate genetic association studies.

## Figures and Tables

**Table 1:** ASSIST coding of data derived from screening for cervical cancer and/or from surveillance of women with precancerous lesions or cervical cancer.

(a) Normal (+ Within Normal Limits)	***Assist code = 0***
(b) Low Grade Cervical Intraepithelial Neoplasia (LCIN)	***Assist code = 1***
(c) High Grade Cervical Intraepithelial Neoplasia (HCIN)	***Assist code = 2***
(d) Invasive Cervical Cancer	***Assist code = 3***

**Table 2:** Mapping between the University of Ghent (U-GENT) Hospital, Bethesda and ASSIST cytology classifications.

U-GENT cytology classification	BETHESDA classification	ASSIST cytology coding
Normal Cervix	Negative for intraepithelial lesion or malignancy	0
Reactive Changes	Reactive changes	0
ASCUS	ASCUS	1
LSIL (CIN1, Coilocytosis, Mild Dysplasia)	LSIL	1
ASC-H	ASC-H	2
HSIL (CIN2, CIN3, CIS, Moderate Dysplasia, Severe Dysplasia)	HSIL	2
SCC (Squamous Cell Carcinoma)	SCC	3
AGC-NOS	Glandular atypical cells (NOS or specify in comments)	1
AGC-favor neoplasia	Atypical glandular cells favor neoplasia	2
AIS	AIS	2
Adenocarcinoma	Adenocarcinoma	3

**Table 3:** Mapping between the Charité Hospital, Munich and ASSIST cytology classifications.

Charité cytology classification	MUNICH classification	ASSIST cytology coding
PAP I	PAP I	0
PAP II	PAP II	0
PAP IIw PAP IIk	PAP IIw	1
PAP III	PAP III	1
PAP IIID LSIL	PAP IIID	1
PAP IVa	PAP IVa	2
PAP IVb	PAP IVb	2
HSIL	PAP IVa or PAP IVb	2
PAP V	PAP V	3

**Table 4:** ASSIST medical rule and procedural logic for CxPCa Severity Index definition.

BEGIN
IF examination result of <Histology> OR <Colposcopy> OR <Cytology> available THEN
IF two or more diagnostic test results exist AND difference between any two results > = 2 THEN
<Severity Index> is defined as the greatest index among the results
ELSE
IF <Histology> definition available THEN
use <Histology> as <Severity Index> definition
ELSE
IF <Colposcopy> definition available THEN
use <Colposcopy> as <Severity Index> definition
ELSE
use <Cytology> definition as <Severity Index> definition
ENDIF
ENDIF
ENDIF
ELSE
<Severity Index> may not be defined
ENDIF
END

## Glossary Terms

AGC: Atypical Glandular Cells
AIS: Adenocarcinoma In Situ
ASC-US: Atypical Squamous Cells of Undetermined Significance
ASC-H: Atypical Squamous Cells favor High grade disease
CIN: Cervical Intraepithelial Neoplasia
CIS: Carcinoma In Situ
CYP1A1: CYtochrome P450
family 1: subfamily A, polypeptide 1
CxPCa: Cervical Precancer and Cancer
DNA: DeoxyriboNucleic Acid
EHR: Electronic Health Record
GSTM1: Glutathione S-Transferase M1
GSTT1: Glutathione S-Transferase Theta 1
HCIN: High grade Cervical Intraepithelial Neoplasia
HCLSIG: (Semantic Web) Health Care and Life Sciences Interest Group
LGSIL: Low Grade Squamous Intraepithelial Lesion
HGSIL: High Grade Squamous Intraepithelial Lesion
LCIN: Low grade Cervical Intraepithelial Neoplasia
MTHFR: Methylene Tetra Hydro Folate Reductase
NOS: Not Otherwise Specified
OWL: Web Ontology Language
PAP: Papanicolaou test
PCR: Polymerase Chain Reaction
SCC: Squamous Cell Carcinoma
SI: Severity Index
W3C: World Wide Web Consortium
